# Growth of 19 conifer species is highly sensitive to winter warming, spring frost and summer drought

**DOI:** 10.1093/aob/mcab090

**Published:** 2021-07-30

**Authors:** Yanjun Song, Ute Sass-Klaassen, Frank Sterck, Leo Goudzwaard, Linar Akhmetzyanov, Lourens Poorter

**Affiliations:** Forest Ecology and Forest Management Group, Wageningen University and Research, AA, Wageningen, the Netherlands

**Keywords:** Conifer species, growth potential, growth sensitivity, phylogeny, spring frost, summer drought, winter temperature

## Abstract

**Background and Aims:**

Conifers are key components of many temperate and boreal forests and are important for forestry, but species differences in stem growth responses to climate are still poorly understood and may hinder effective management of these forests in a warmer and drier future.

**Methods:**

We studied 19 Northern Hemisphere conifer species planted in a 50-year-old common garden experiment in the Netherlands to (1) assess the effect of temporal dynamics in climate on stem growth, (2) test for a possible positive relationship between the growth potential and climatic growth sensitivity across species, and (3) evaluate the extent to which stem growth is controlled by phylogeny.

**Key results:**

Eighty-nine per cent of the species showed a significant reduction in stem growth to summer drought, 37 % responded negatively to spring frost and 32 % responded positively to higher winter temperatures. Species differed largely in their growth sensitivity to climatic variation and showed, for example, a four-fold difference in growth reduction to summer drought. Remarkably, we did not find a positive relationship between productivity and climatic sensitivity, but instead observed that some species combined a low growth sensitivity to summer drought with high growth potential. Both growth sensitivity to climate and growth potential were partly phylogenetically controlled.

**Conclusions:**

A warmer and drier future climate is likely to reduce the productivity of most conifer species. We did not find a relationship between growth potential and growth sensitivity to climate; instead, some species combined high growth potential with low sensitivity to summer drought. This may help forest managers to select productive species that are able to cope with a warmer and drier future.

## INTRODUCTION

Climate change is leading to increased warming and an increased frequency of late spring frosts and summer droughts (Inouye, 2000; Hartmann, 2011), with potentially large repercussions for forest and tree productivity (Ciais *et al.*, 2005; Gazol *et al.*, 2019). An improved understanding of how trees respond to long-term climatic variation may allow for a better understanding of under what conditions forests are a net carbon source (Kurz *et al.*, 2008) or a carbon sink (Walker *et al.*, 2021). Here we focus on the effects of climate on stem growth of conifer species, which dominate large areas in cold boreal forests, temperate forests and dry Mediterranean forests (Farjon and Filer, 2013), and account for nearly one-third to the global forest carbon stock (Pan *et al.*, 2011).

### Climate–growth relationships of conifer species

Dendrochronological studies provide a long-term perspective on how stem growth responds to climate (Fritts, 1976; Babst *et al.*, 2013; Charney *et al.*, 2016). Such growth responses for conifer species have been studied on a macro-scale across continents with climate gradients (Williams *et al.*, 2010; Klesse *et al.*, 2018). Winter temperature, spring frost and summer drought are primary factors limiting growth (D’Orangeville *et al.*, 2016; Julio Camarero *et al.*, 2018; Vitasse *et al.*, 2019). Generally, in cold and mild areas, high temperatures in winter and early spring benefit evergreen conifer species’ growth because species still maintain photosynthesis (Larcher, 2000). Warmer conditions during early spring and warming of frozen soils may lead to an earlier start of the growing season (Williams *et al.*, 2015; Harvey *et al.*, 2020), which increases the length of the growing season and tree growth. Yet, early warming and increased tree cambial and bud activity may also enhance the risk of damage by late spring frosts and, thus, reduce growth (Dy and Payette, 2007; Harvey *et al.*, 2020). In temperate regions, especially arid regions (Gazol *et al.*, 2018), high summer temperatures can lead to drought stress, reduced carbon gain, and an early cessation of the growing season (Choat *et al*., 2018; Dietrich *et al.*, 2019). In contrast, in cold regions, higher summer temperatures may positively affect tree growth (Klesse *et al.*, 2018).

From these studies and others (Babst *et al.*, 2013; Bhuyan *et al.*, 2017; Montwé *et al.*, 2018), it is difficult to draw generalizations about differences in species-specific responses to climate, because different species are compared across different parts of the climatic gradient. Moreover, in general only one or a few conifer species have been included in these studies (Vitali *et al.*, 2017; Julio Camarero *et al.*, 2018; Klesse *et al.*, 2018). To better understand species differences in climatic response, long-term common garden experiments can control the potentially confounding effects of climatic or soil conditions on the growth of different tree species (cf. Huang *et al.*, 2017).

### Relationship between growth sensitivity and potential

Growth sensitivity is defined as a large plastic response in growth in response to climatic variation (Klesse *et al.*, 2018). Climatic growth sensitivity varies across species because of differences in species traits. For example, deep roots enable species to take up water from deeper soil layers during drought (Huang, 2000). Some traits may lead to fast growth and also high growth sensitivity. For example, species with wide tracheids have a high potential growth rate, which normally comes at the cost of hydraulic safety because cheap light wood has a lower cell wall reinforcement (low thickness to span ratio) (Pittermann *et al.*, 2006; Sperry *et al.*, 2006). Wide tracheids also allow for high hydraulic conductivity and, hence, high gas exchange, photosynthesis and growth rates (Chave *et al.*, 2009). Simultaneously, these species would also be sensitive to frost and drought, because increased conduit size increases species vulnerability to freezing- and drought-induced cavitation (Mayr *et al.*, 2006). However, few studies have directly evaluated these relationships between growth potential and growth sensitivity to climatic variation.

In this study, we compared stem-growth responses across 19 conifer species from the Northern Hemisphere, which were planted in the late 1960s in a common garden experiment in the Netherlands. We used tree-ring analysis to evaluate how annual stem growth responded to variation in climatic factors over 44 years. It is difficult to find common garden experiments old enough to assess species differences in long-term growth responses, as done here on 19 conifer species growing under Dutch maritime climate conditions.

First, we identified which climatic factors limit annual stem growth of the species in different months and seasons before and during the growing period. We hypothesized that growth of the 19 species is reduced by spring frosts and summer droughts (e.g. via high temperature and low precipitation) and sensitive to warm winters. Specifically, spring frost could limit growth because of freezing-induced cavitation, or damage to leaf and twig cambial activity (Begum *et al.*, 2010; Li *et al.*, 2013), which can delay the start of the growing season. Summer droughts reduce growth due to lower stomatal conductance reducing photosynthetic rates (McDowell *et al.*, 2008). Warm winters can lead to higher stem growth due to increased photosynthetic activity in warm areas (Fry and Phillips, 1977), but can also reduce growth through increased respiration (Larsen *et al.*, 2007) or drought stress due to water deficit (DeSoto *et al.*, 2014).

Second, we quantified species differences in growth potential and related it to their growth sensitivity to climatic variation. We predicted a positive relationship between stem growth potential and growth sensitivity to climatic variation, because the traits that lead to high inherent growth rates, such as large cells with thin cell walls that facilitate high metabolic activity, may lead to a higher sensitivity (e.g. by a higher cavitation vulnerability) to extreme climate events such as late spring frosts and summer droughts.

Third, we evaluated to what extent the growth sensitivity of species to climatic factors is phylogenetically controlled. Given that closely related conifer species are adapted to different environmental conditions (Zanne *et al.*, 2014), we expect that growth potential and stem growth sensitivity to climate is only weakly phylogenetically controlled.

## MATERIAL AND METHODS

### Study site

This study was carried out at Schovenhorst Estate (52.25°N, 5.63°E), east of Putten, the Netherlands. The elevation is ~30 m above sea level. The climate is characterized as mild maritime with a mean annual temperature of 10.1 °C, maximum annual temperature of 13.5 °C, minimum annual temperature of 6.0 °C and a mean annual rainfall of 830 mm averaged over 44 years (1974–2017). Precipitation is quite evenly distributed across seasons (Supplementary Data Fig. S1). Soils are derived from postglacial loamy sand deposits, forming well-drained and acidic (pH ~4) podzolic soils of generally low fertility (Cornelissen *et al.*, 2012; van der Wal *et al.*, 2016). The groundwater table is below 19.04 m and considered not accessible by trees (TNO-NITG, 2020).

### Sample design and species selection

We used a long-term established common-garden experiment established between 1916 and 1974 (Table 1), which has advantages to assess the long-term climate–growth relationship, but has also shortcomings such as no or limited control on the design of the experiment (e.g. blocks to correct for micro-site-related differences, planting dates, plant density, etc). Yet from (limited) historical information and the actual situation, we can infer that trees have been planted in groups per species and with considerable distance to each other to exclude strong resource competition. The stands were never managed. Most species were planted in monospecific stands. This experiment was initially established to select non-native species with high timber production potential for the Netherlands, and only one native species was included (*Taxus baccata*) (Willinge Gratama-Oudemans, 1992). In our study, 19 coniferous species were selected, including genera and species originating from different biogeographical zones (Table 1). For each species, ten dominant and healthy individuals were selected that formed part of the canopy and hence could express their full growth potential.

**Table 1.** Overview of the 19 study species, their distribution area, the growth period considered, the average stem diameter at breast height (dbh) of the sampled trees (*N* = 10 per species) in 2017/2018, and the mean basal area increment (BAI). The standard deviation is shown in parentheses. Mean BAI and s.d. are calculated based on tree-ring data (Supplementary Data Fig. S2). Distribution areas are derived from Farjon and Filer (2013)

Species	Distribution areas	Species abbreviations	Period considered	dbh (cm)	Mean BAI (cm^2^ yr^–1^)
*Abies alba*	Europe	ABAL	1958–2018	46.9 (10.3)	29.5 (5.2)
*Abies grandis*	North America	ABGR	1940–2017	76.9 (8.7)	55.9 (6.1)
*Abies veitchii*	Northern Honshu, Japan	ABVE	1979–2018	27.1 (3.1)	12.6 (1.6)
*Chamaecyparis lawsoniana*	North America (USA)	CHLA	1911–2017	48.2 (9.6)	20.0 (2.4)
*Cryptomeria japonica*	Eastern Asia	CRJA	1969–2018	35.6 (6.2)	18.3 (1.9)
*Larix kaempferi*	Eastern Asia	LAKA	1945–2018	49.4 (6.8)	23.1 (3.2)
*Pinus armandii*	Eastern Asia	PIAR	1981–2018	24.0 (3.2)	10.9 (1.5)
*Pinus nigra*	South-eastern Europe	PINI	1945–2018	44.6 (6.5)	19.3 (1.5)
*Picea abies*	Europe	PIAB	1969–2018	43.4 (5.2)	27.7 (3.9)
*Picea omorika*	Europe	PIOM	1953–2018	30.9 (30.9)	13.7 (1.1)
*Picea orientalis*	Mainland Asia	PIOR	1944–2017	47.7 (8.1)	23.8 (2.3)
*Picea sitchensis*	North America	PISI	1972–2018	44.5 (8.8)	34.8 (4.9)
*Pseudotsuga menziesii*	North America	PSEM	1916–2017	76.3 (10.1)	48.6 (4.3)
*Taxus baccata*	Europe	TABA	1957–2018	31.2 (9.9)	9.8 (1.6)
*Taxus cuspidata*	Mainland Asia	TACU	1974–2018	14.8 (4.0)	3.9 (0.4)
*Thuja plicata*	North America	THPL	1942–2017	74.3 (12.7)	47.0 (5.9)
*Tsuga canadensis*	North America	TSCA	1972–2018	31.6 (4.8)	18.4 (2.1)
*Tsuga diversifolia*	Japan	TSDI	1972–2018	25.3 (3.6)	9.3 (1.7)
*Tsuga heterophylla*	North America	TSHE	1971–2017	53.2 (6.4)	42.6 (4.6)

### Tree-ring analysis

To investigate stem growth rates and annual growth variation, we took two incremental cores from each of the ten selected individuals per species at two opposite sides at 1.3 m stem height using Haglof Pressler increment borers. All samples were cut with a microtome (Gärtner and Nievergelt, 2010) and sanded with progressively finer sandpaper (grain sizes from P600 to P1000, Fepa Abrasives) to improve the visibility of tree-ring boundaries. Flat surfaces were subsequently scanned at 2000 dpi using an Epson scanner (Epson 10000XL). Tree-ring width (TRW) was measured, and time series were cross-dated to assign a calendar year to each ring using CooRecorder and CDendro (v. 9.0, Cybis Electronik and Data AB, Sweden). Cross-dating was done by first matching the ring-width patterns of individual trees, and then different trees of the same species. To better detect the effect of climate on tree growth, we removed the confounding impact of tree age on ring-width. For each individual, TRW series were detrended with the R ‘*dplR*’ package (Bunn, 2008). We first fitted a cubic smoothing spline with a 50 % frequency cutoff at 15 years. This standardization is crucial for assessing the climate–growth relationship, as it allows us to remove all the low-frequency variation (i.e. non-climatic noise) (Cook *et al.*, 1990). We then divided the raw TRW value with the corresponding year’s spline value, thus obtaining dimensionless ring-width index time series (TRI) per tree. TRI chronologies were calculated for each species by averaging (biweight robust mean) the detrended individual time series of trees of the same species using the ‘*dplR*’ package (Bunn, 2008). Chronology calculation strengthens the common climatic signal in the tree populations by at the same time dampening individual tree variation (Cook *et al.*, 1995). For more information about tree-ring characteristics, see Supplementary Data Table S1.

### Climate data from the Netherlands

To check how annual climatic factors affected species growth, climate records were retrieved from the weather station ‘De Bilt’, situated ~45 km from the study site (KNMI, https://www.knmi.nl/home; https://www.knmi.nl/nederland-nu/klimatologie/daggegevens).

To evaluate how spring frost days affected stem growth, the number of spring frost days was defined as days where the minimum daily temperature dropped below 0 °C (Chudnovskii, 1949; Gurskaya, 2014). Monthly frost days were then aggregated by counting the number of frost days per month. Because there were nearly no frost days recorded in May, we considered March and April for calculating spring frost days.

To assess the effects of summer droughts (i.e. water availability during summer) on growth of the selected species, monthly data of mean temperature (°C) and total precipitation (mm per month) were downloaded (KNMI, https://www.knmi.nl/home). We also calculated the standardized precipitation evapotranspiration index (SPEI), which is an indicator for the climate–water balance and a proxy for water availability (Vicente-Serrano *et al.*, 2010). For this calculation, we first calculated PET using the Thornthwaite method, which required monthly mean temperature and latitude (Thornthwaite, 1948). Next, we calculated SPEI based on precipitation (P) and PET (see Vicente-Serrano *et al.*, 2010). SPEI was a standardized index with an average value of 0 and a standard deviation of 1. SPEI can be calculated at different time scales. To focus on summer drought, we aggregated the climate–water balance based on a 3-month scale from June to August (Vicente-Serrano *et al.*, 2010). SPEI was calculated using the R ‘SPEI’ package software (Beguería *et al*., 2017).

### Data analysis

To analyse climate–growth relationships, the common period 1974–2017 was selected to compare the growth responses across species. Since *Pinus armandii* and *Abies veitchii* included individuals established after 1974, tree rings of these two species were analysed from 1981 to 2017 (Table 1). To avoid the inflation of significant correlations by calculating many for a given question (Biondi, 1997), we used 1000 bootstrapped subsets for each species from the climate and TRI data to calculate correlation coefficients between TRI chronologies and the monthly climatic factors. We showed such correlations with TRI with climate data from June in the year preceding the tree-ring formation until September in the year of tree-ring formation (Biondi and Waikul, 2004).

To evaluate and specify the growth sensitivity to different climatic factors, we selected climate conditions from different seasons that were hypothesized to be growth-limiting, namely frost in spring (the number of frost days from March 1 to April 30), summer water availability (i.e. SPEI averaged from 1 June to 31 August) and winter temperature (mean temperature from 1 January to 31 March). Climate–growth analyses for the 19 species were carried out for the common period (1974–2017). The climatic effects on stem growth (i.e. TRI) were assessed using a linear mixed model, with the averaged tree-ring width index from two cores per tree as the dependent variable, the interactions between seasonal climatic variables and species as fixed factors, and individuals as random factors. The variance inflation factor (VIF) was <5 for all climatic factors, indicating that there were no collinearity problems with the predictor variables (Gould *et al.*, 2016). To compare the effect sizes, climatic data were standardized before analysis by subtracting the mean and dividing it by the standard deviation, which also improved the homogeneity and normality of residuals. To check homogeneity and normality, residuals with a Q-Q plot and frequency plot were produced (Supplementary Data Figs S2 and S3). We used 7747 annual rings as data points rather than 8360 (44 years × 19 species × 10 trees) because parts of the cores were damaged for some individuals and outliers were removed. Regression coefficients were used to describe the stem growth sensitivity of each species to four climatic factors: spring frost, summer drought in the year of stem growth, summer drought in the year preceding growth and winter temperature, respectively. To obtain the proportional variance (*R*^2^) explained by the multiple regression, species-specific *R*^2^ was calculated from the square of correlations between the predicted TRI (based on the multiple regression in Table 2) and the observed TRI both on the individual and mean chronology level (see Table S2). Additionally, the standard deviation of TRI was used as an indicator of growth sensitivity, because it captures the overall annual variation in growth that might be attributed to all climatic factors, and it is therefore an indicator of the overall climate sensitivity of a tree species. All models were implemented with the package ‘nlme’ (Pinheiro *et al.*, 2017) in the R statistical environment (R Core Team, 2019).

**Table 2.** Results of a species-specific multiple regression analysis of stem growth (i.e. tree-ring index) of 19 coniferous tree species on five selected environmental variables: number of spring frost days, summer SPEI from June to August (i.e. the standardized precipitation evapotranspiration index) in the current years, summer SPEI in the previous year and winter temperature. Regressions were based on the common period from 1974 to 2017. Standardized regression coefficients are shown. Coefficients in bold refer to statistically significant relationships (*P *< 0.05). Linear mixed effect models were used to explain stem growth, using interaction terms between species and environmental variables as fixed factors, and individual as a random variable. *R*^2^m is the marginal and *R*^2^c is the conditional *R*^2^. Mixed models have the same *R*^2^m and *R*^2^c because random effects nearly explained zero in the model. For species-specific *R*^2^, see Supplementary Data Table S2

Species	Spring frost	Current summer SPEI	Previous summer SPEI	Winter temperature
*Abies alba*	−0.012	**0.040**	0.012	0.006
*Abies grandis*	−0.017	0.020	0.012	**0.042**
*Abies veitchii*	−0.004	0.024	−0.019	0.007
*Chamaecyparis lawsoniana*	−**0.039**	**0.028**	0.023	0.023
*Cryptomeria japonica*	0.014	**0.050**	**0.033**	**0.125**
*Larix kaempferi*	−**0.121**	**0.052**	**0.102**	**−0.043**
*Pinus armandii*	−0.020	**0.044**	0.004	−0.021
*Pinus nigra*	−0.021	**0.053**	−0.017	0.016
*Picea abies*	−**0.055**	**0.077**	0.003	**−0.038**
*Picea omorika*	−**0.081**	**0.064**	**0.026**	**−0.042**
*Picea orientalis*	−**0.057**	**0.050**	**0.044**	0.018
*Picea sitchensis*	−**0.050**	**0.061**	0.008	**−0.040**
*Pseudotsuga menziesii*	−**0.054**	**0.032**	0.017	**0.032**
*Taxus baccata*	−0.003	**0.077**	−0.017	**0.103**
*Taxus cuspidata*	0.028	**0.047**	−0.020	**0.053**
*Thuja plicata*	−0.023	**0.046**	0.002	−0.005
*Tsuga canadensis*	−0.027	**0.038**	−0.018	−0.008
*Tsuga diversifolia*	0.011	**0.066**	−**0.029**	−0.028
*Tsuga heterophylla*	−0.017	**0.049**	−0.020	**0.057**
*R*^2^m/*R*^2^c	0.129/0.129			

To quantify the stem growth potential of species, we calculated for each tree the slope between the estimated stem radius calculated from added annual ring widths and tree age for the first 20 years (i.e. stem diameter growth, mm yr^–1^). We restricted this analysis to the first 20 years because in this period the canopy of the stand was probably relatively open, resulting in a relatively linear relationship during this period, which became lost over longer periods (Supplementary Data Fig. S4a, b). Similar analyses using basal area increment were also shown (Fig. S4c, d). The slopes were calculated by running a linear mixed effect model, using cumulative annual ring width as the response variable, the corresponding age of that ring and species as fixed factors, and the slope between corresponding age and individual as random coefficients (Saunders and Wagner, 2008). This random slope linear mixed model allows tree ring variables associated with the individual trees to predict changes in coefficients that generate smoothed estimates of annual radial growth over time (McLane *et al.*, 2011). *Pseudotsuga menziesii* was not included in the model because we did not take the samples to the pith; thus, we cannot determine the first 20 years during their growth period. Instead, the cumulative ring width data were used when the first 20 years were available. The linear regression used cumulative ring widths as a dependent factor, year as a fixed factor and individual as random factors. Hence, the slope of the regression was used to estimate the growth potential for *P. menziesii*. Given that biomass may reflect real growth and stem area growth (BAI) may indicate size growth and timber volume production, the averaged stem area growth (cm^2^ yr^–1^, Table 1) and stem mass growth (kg yr^–1^/m) were also provided as alternative proxies of growth potential. Stem mass growth was calculated by multiplying stem area growth (m^2^ yr^–1^) and wood density (kg m^–3^), and reflect the biomass increment per metre stem length (Sterck *et al.*, 2012).

To estimate to what extent growth sensitivity and growth potential are phylogenetically controlled, we calculated Blomberg’s *K* (Blomberg *et al.*, 2003) using the ‘phytools’ package (Revell, 2012) in R (R Core Team, 2019). Blomberg’s *K* assumes a Brownian motion of evolution, where temperature leads to random mutations and changes in the genome. *K* values compare the observed phylogenetic signal in a trait to traits under a Brownian motion of trait evolution. *K* is calculated as the quotient of observed and expected ratios of mean square errors (MSE) (eqn 1). The observed ratio is the MSE_0_ of the tip data from the phylogenetically correct mean, divided by the MSE of the data calculated using the variance–covariance matrix derived from the tree. The expected ratio is the expected variation under Brownian motion relative to the number of taxa in the phylogeny (Blomberg *et al.*, 2003; Adams, 2014).


$$\begin{array}{l} K=observed\frac{MSE_{0}}{MSE}/expected\frac{MSE_{0}}{MSE} \end{array}$$ (1)

*K* values may range from 0 (the null expectation) to infinity. *K* values around 1 indicate that there is a significant phylogenetic signal as expected under the Brownian motion model; *K* values lower than 1 indicate that the trait is less phylogenetically controlled than expected; *K* values closer to 0 indicate that the trait is not phylogenetically controlled (i.e. that the trait has evolved independently of phylogeny); and *K* > 1 indicates the trait is strongly phylogenetically conserved (CaraDonna and Inouye, 2015). The statistical significance of *K* values was tested followed the method of Kembel *et al.* (2010).

## RESULTS

### General climate–growth relationships

**Fig. 1.**
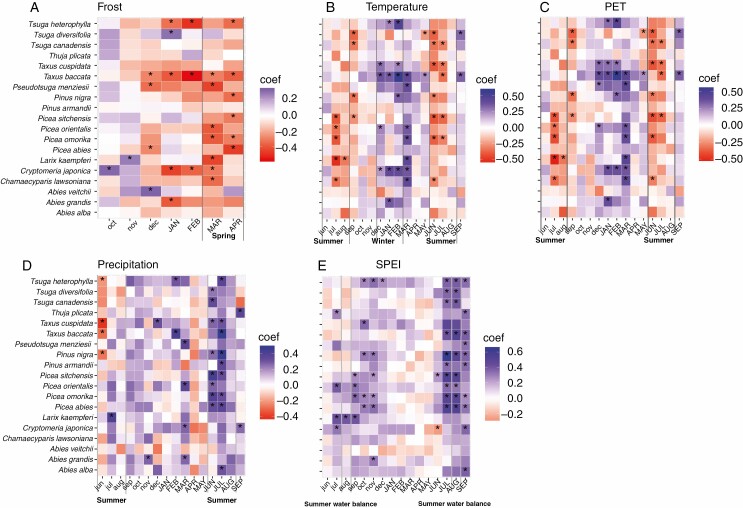
Bivariate bootstrapped correlation coefficients between tree-ring width index (TRI) and climate variables were calculated for 19 coniferous species (in rows) and monthly climate data (in columns) for (A) the number of frost days, (B) mean temperature, (C) potential evapotranspiration (PET), (D) precipitation and (E) standardized precipitation evapotranspiration index (SPEI). Correlations are made between TRI and monthly climatic conditions from previous year June (jun) to current year September (SEP). For 17 species correlations were made across a 44-year period from 1974 to 2017 (*N* = 44 per species), whereas correlations for *P. armandii* and *A. veitchii* were based on a 37-year period from 1981 to 2017 (*N* = 37 per species). The standardized precipitation-evapotranspiration index (SPEI) is calculated over a 3-month period (i.e. SPEI AUG reflects water availability from June to August). Lower-and upper-case letters represent monthly climate data from last year and current year for tree-ring formation, respectively. Blue/purple cells indicate positive correlations, red/orange cells indicate negative correlations, and asterisks indicate significant (*P *< 0.05) correlations.

For all 19 conifer species studied, annual stem growth variation was expressed by TRI and correlated with multiple climatic factors (Fig. 1). TRI of 37 % of the conifer species was significantly negatively affected by the number of spring frost days in March and April (Fig. 1A, Table 2). At the same time, the growth of 32 % of the species was significantly positively correlated with warmer conditions during the winter months December, January and February (Fig. 1B, Table 2). *Cryptomeria japonica*, *Tsuga heterophylla* and *Taxus baccata* were most sensitive in their stem growth response to cold winters (Table 2). During summer, the correlation between growth and temperature became generally negative (Fig. 1B). Yet, species show different trends in sensitivity to cold winters and to dry summers. Growth was positively correlated with variables reflecting a positive water balance (i.e. high SPEI values and precipitation, low summer temperature and PET) (Fig. 1C–E), which indicates that water availability during summer was limiting stem growth in >50 % of the conifer species, with the strongest negative impact for the *Picea* species, *Pinus nigra* and *Taxus baccata* (Fig. 1E, Table 2). In all species annual stem growth increased significantly with summer SPEI (and this was significant for 89 %), that is with water availability in June, July and August, and with summer SPEI of the preceding year (21 % of the species, Table 2). The large absolute values of the standardized regression coefficients indicate that species growth is generally highly sensitive to climatic variation. Yet, it also became apparent that the three *Abies* species seem to be less responsive to, in particular, spring frost and water availability during summer compared to the rest of the conifer species.

### Relationship between stem growth potential and growth sensitivity to climate

**Fig. 2.**
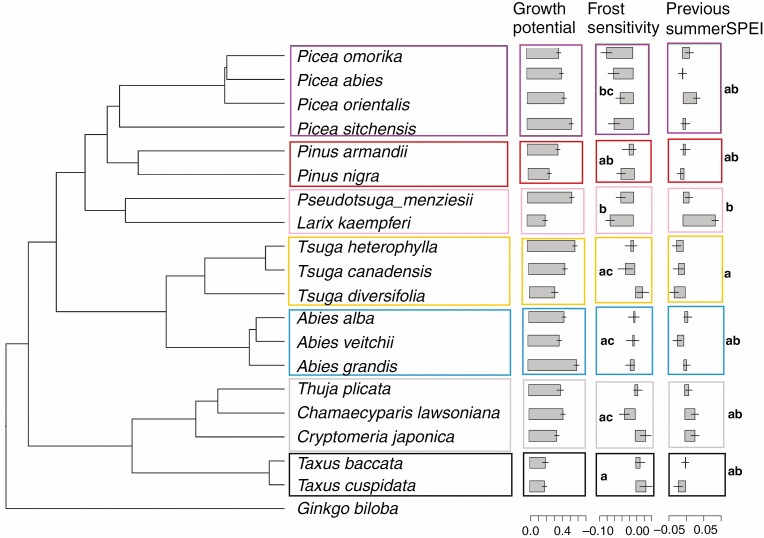
A visualization of the significant phylogenetic signals with stem growth sensitivity to climate and growth potential (see Table 4): growth potential refers to the stem growth rate (cm yr^–1^) during the first 20 years for 19 species; frost sensitivity refers to the stem growth response to number of frost days in March and April; and Previous summer SPEI refers to the stem growth sensitivity to summer SPEI in the previous year. The effects are shown as bar plots corresponding to the tips of the phylogeny. *Ginkgo biloba* was selected as an outlier and reference. Molecular phylogeny is from Zanne *et al.* (2014). Different colours indicate different genera. Significant differences are also tested between genera and significant differences are shown by using different letters. The horizontal line is the error bar.

**Fig. 3.**
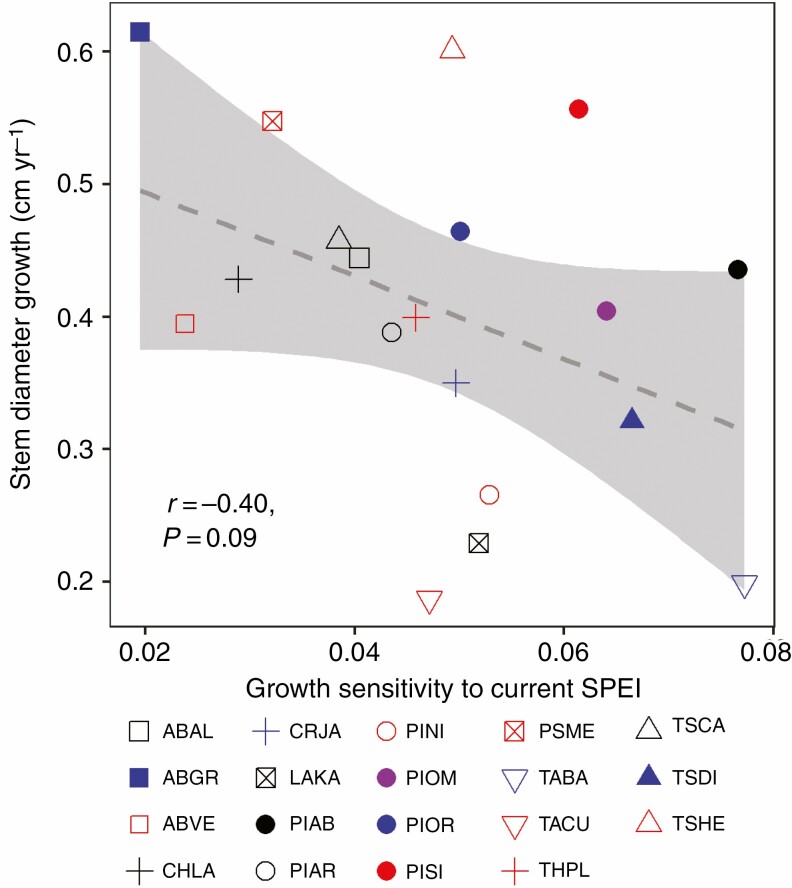
Relationships between stem diameter growth (cm yr^–1^) and growth sensitivity to summer SPEI of current year. Regression lines and 95 % confidence intervals (grey), Pearson correlation coefficients (*r*) and *P*-values are shown. For species abbreviations see Table 1.

Among three proxies for growth potential (i.e. stem diameter growth, stem mass growth and stem area growth), stem diameter growth rate varied >3-fold across species, with *Abies grandis* being the fastest growing species (0.61 cm diameter growth per year) and species from the genus *Taxus* the slowest growing species (*Taxus baccata*: 0.20 cm per year, *Taxus cuspidata* 0.19 cm per year, Fig. 2); similar trends were found for stem mass growth and stem area growth, and both of them varied >10-fold across species, ranging from *Taxus cuspidata* (0.23 kg yr^–1^ m^–1^, 3.91 cm^2^ yr^–1^, Supplementary Data Fig. S5b-c) to *Abies grandis* (2.94 kg yr^–1^ m^–1^, 55.90 cm^2^ yr^–1^, Fig. S5b-c). Unexpectedly, there were no positive relationships between growth potential and growth sensitivity to spring frost (Table 3). Instead, only one proxy of growth potential (i.e. stem diameter growth) was negatively correlated with stem growth sensitivity to current summer SPEI, although this was only marginally significant (Table 3, *r* = −0.40, *n* = 19, *P* = 0.09; Fig. 3). The correlation was mainly driven by *Abies grandis* (high growth and low sensitivity to summer SPEI) and the opposite combination was shown by *Taxus baccata* (i.e. low growth and high sensitivity).

**Table 3.** Pearson correlations between stem growth sensitivity and growth potential of conifer species (*N* = 19 species). Growth potential refers to stem diameter growth (cm yr^–1^), stem area growth (cm^2^ yr^–1^), stem mass growth (kg yr^–1^ m^–1^) and stem standardized (Std.) area growth (cm^2^ cm^–1^ yr^–1^). The growth sensitivity to spring frost days, summer SPEI (i.e. the standardized precipitation evapotranspiration index) in the current years, summer SPEI in the previous year and winter temperature was quantified using the multiple regression coefficients of Table 2. Standard deviation (s.d.) for tree ring index (TRI) from time series was also used as growth sensitivity. Correlation coefficient (*r*) and *P*-value (*P*) are shown

Growth sensitivity	Stem growth potential							
	Diameter growth		Area growth		Mass growth		Std. area growth	
	*r*	*P*	*r*	*P*	*r*	*P*	*r*	*P*
Spring frost	−0.16	0.52	−0.23	0.35	−0.24	0.33	0.05	0.84
Current SPEI	−0.40	0.09	−0.37	0.12	−0.29	0.23	−0.01	0.98
Previous SPEI	−0.03	0.91	0.16	0.51	0.13	0.60	0.07	0.78
Winter temperature	−0.12	0.63	−0.01	0.97	−0.03	0.90	0.11	0.65
s.d. (TRI)	−0.22	0.36	0.10	0.68	0.04	0.86	0.07	0.78

**Table 4.** Phylogenetic signal of growth sensitivity to spring frost, summer drought, winter temperature and growth potential for 19 conifer species. Growth potential refers to stem diameter growth (cm yr^–1^), stem area growth (cm^2^ yr^–1^), standardized stem area growth (cm^2^ cm^–1^ yr^–1^), and stem mass growth (kg yr^–1^ m^–1^). Significant values (*P *< 0.05) are shown in bold, and indicate that growth sensitivity is phylogenetically conserved

Phylogenetic trait	Blomberg’s	
	*K-*value	*P*-value
Growth sensitivity to spring frost	**0.87**	0.003
Growth sensitivity to previous SPEI	**0.78**	0.008
Growth sensitivity to current SPEI	0.34	0.39
Growth sensitivity to winter temperature	0.44	0.17
Stem diameter growth during whole period	0.27	0.67
Stem diameter growth for the first 20 years	**0.55**	0.04
Stem area growth	0.28	0.59
Standardized stem area growth	0.20	0.90
Stem mass growth	0.25	0.70

### The phylogenetic signal on stem growth sensitivity to climate and growth potential

Few significant phylogenetic signals were observed for stem growth sensitivity to climatic factors. Only stem growth sensitivity to spring frost (*K* = 0.87, *P *< 0.01) and previous summer SPEI (*K* = 0.78, *P*< 0.01) showed significant phylogenetic signals (Table 4). This was also supported by ANOVAs that showed that some main phylogenetic groups differed in their stem growth sensitivity to spring frost (ANOVA, *F*_6,12_ = 6.11, *P* = 0.004): the Taxaceae, Cupressaceae (*Chamaecyparis*, *Thuja* and *Cryptomeria*) and *Abies* were less sensitive to spring frost compared to *Pseudotsuga* and *Larix* (Tukey’s post-hoc test, *P*< 0.05) (Fig. 2). The phylogenetic groups also differed in their sensitivity to previous summer SPEI (ANOVA, *F*_6,12_ = 3.63, *P* = 0.03); *Pseudotsuga* and *Larix* were more sensitive in stem growth responses to previous summer SPEI compared to *Tsuga* species (Table 2, Fig. 2). Stem diameter growth was significantly phylogenetically conserved (*K* = 0.55, *P* = 0.04) (Table 4), although the main genera/phylogenetic groups did not differ significantly in their stem diameter growth (ANOVA, *F*_6,12_ = 2.08, *P* = 0.13), whereas stem area growth and stem mass growth did not show any phylogenetic signal (Table 4). Overall, these phylogenetic trends were relatively weak, indicating that stem growth sensitivity to climatic factors and growth potential are relatively labile.

## DISCUSSION

We analysed how 19 conifer species that were planted in a common garden experiment and growing under a mild maritime climate differed in their growth responses to winter temperature, spring frost and summer drought over 44 years. Almost all species showed a significant reduction in their stem growth in response to summer droughts, but the magnitude of this response varied four-fold across species (Table 2). About one-third of the species showed the expected reduced stem growth with more frost days during spring (37 %), or increased stem growth with a higher mean winter temperature (32 %). We found no positive relationship between stem growth potential and growth sensitivity to climate across species (Table 3). Here, we will discuss how these selected climatic factors and phylogeny affect the annual variation in stem growth of conifer species, and the possible implications of climate change for the future productivity and resilience of conifer species and forests.

### Summer droughts

It was expected that even under mild maritime conditions, with an on average evenly distributed rainfall regime, summer droughts would reduce stem growth of nearly all conifer species. Most of the studied conifer species (89 %) indeed showed reduced stem growth in response to summer droughts during the growing season and, to a lesser extent (32 %), during the previous growing season (Table 2). Summer droughts were expected to reduce stem growth because trees avoid excessive water by closing their leaf stomata, resulting in reduced gas exchange, photosynthetic carbon gain and ultimately also stem growth (Sala *et al*., 2010; Adams *et al*., 2017). For similar climate conditions, such growth rate reductions were also observed for broadleaved species (Weemstra *et al.*, 2013). The effect on stem growth may also result from low stem water potentials that come with dry conditions, hamper cell division and cell expansion, and limit stem growth (Cuny and Rathgeber, 2016). Alternatively, trees may shift their allocation of carbohydrates from stem growth towards the formation of new roots for increasing water uptake (Oberhuber *et al.*, 2017; Markesteijn and Poorter, 2009; Huang *et al.*, 2018), or towards the storage of non-structural carbohydrates (NSCs) to facilitate future growth when environmental conditions are more benign (Piper *et al.*, 2017).

The growth sensitivity to summer droughts expressed by the regression coefficients varied four-fold across species (Table 2). *Picea abies* and *Tsuga diversifolia* were amongst the most drought-sensitive species. Picea abies had reduced growth even during mild drought (Bottero *et al*., 2021). These species may possess shallow roots that only absorb water from the topsoil (Schmid and Kazda, 2001; Bolte and Villanueva, 2006; Takahashi and Obata, 2014), and therefore face high risks of encountering dry conditions during rainless periods. Another drought-sensitive species was *Larix kaempferi* (Table 2). *Larix kaempferi* is a winter deciduous conifer and has the largest tracheid size (i.e. 16.0 ± 0.4 µm), thinnest walls (2.1 ± 0.2 µm), highest specific leaf area (120.3 ± 6.0 cm^2^ g^−1^) and lowest leaf density (0.2 ± 0.03 g cm^−3^) among the 19 conifer species in our study (Y. Song *et al*., unpubl. data). These acquisitive traits contribute to fast carbon gain and photosynthesis but come at the expense of high water loss and hence a reduced cavitation resistance to drought (Chave *et al.*, 2009). The two least drought-sensitive species were *Abies grandis* and *Abies veitchii* (Table 2). Their weak response to summer drought could be related to their timing of cambial activity with high growth rates early in the growing season (Cuny *et al.*, 2012), because the effects of drought were largest when droughts occurred during the stem growing season peaks (D’Orangeville *et al.*, 2018). The cambial activity of these two species deserves further study. Additionally, the deep root system of *Abies grandis* probably may allow the species to acquire water from deeper soil layers during dry periods and be less drought-sensitive (Xu *et al.*, 1997). The fact that species showed a four-fold difference in stem growth sensitivity to summer drought means that species selection is key to create future forests that are more drought-resilient under future climate change.

### Winter temperature and late spring frost

The 19 conifer species differed in their stem growth responses to winter temperature. For six out of the 19 study species (32 %), we observed that warmer winter conditions significantly favoured stem growth in the following growing season. The relationship was particularly strong for *Cryptomeria japonica* and *Taxus baccata* (Table 2). These species probably increase metabolic rates at higher winter temperatures and thus extend the length of the growing season, by either increasing photosynthesis facilitating plant growth or benefitting from stored carbohydrates fuelling stem diameter growth during favourable winter temperatures (Huang *et al.*, 2010; D’Orangeville *et al.*, 2016; Puchi *et al.*, 2020). However, four of the 19 species (21 %), namely *Larix* and three *Picea* species, showed the opposite response as they reduced stem growth with higher winter temperatures (Table 2). These four species were also negatively affected by spring frosts (Table 2). Possibly, for these species early growth initiation during higher winter temperatures creates more risk of being injured by spring frosts (Vitasse *et al.*, 2019), which then ultimately leads to the observed negative effects of higher winter temperatures for these four species. Additionally, the shallow-rooted *Picea* species (Blackwell *et al.*, 1990; Schmid and Kazda, 2001; Park *et al.*, 2012) may experience water shortage during transpiration when the topsoil is frozen. These divergent effects of winter conditions are remarkable. However, the mechanisms behind the observed patterns remain speculative and require further in-depth study (e.g. Eysteinsson *et al.*, 2009). Nevertheless, our results indicate that high winter temperature affects conifer stem growth, but in contrasting ways for different species.

Seven out of the 19 species (37 %) reduced growth with an increasing number of spring frost days (Fig. 1A, Table 2), although the majority of species did not show this expected response. In particular, *Picea omorika* and the deciduous *Larix kaempferi* were sensitive to spring frost. Reduced stem growth with spring frost have also been found for trees in other temperate lowland coniferous forests (DeSoto *et al.*, 2014; Shestakova *et al.*, 2016). Normally the effects of spring frost on growth are quantified by the degree-days >5 °C until last frost <−2 °C, since damaging frost events are more harmful after prolonged warm periods (Vitasse *et al.*, 2019). The mild maritime climate in our study area makes it difficult to use the same method as Vitasse *et al.* (2019) to quantify such frost days, whereas the frost days (minimum temperature <0 °C) in this study still physiologically affect stem growth.

We did not find any tissue damage at the beginning of the tree rings, which indicates that cell formation either had not yet started or was well underway. Yet, we found tissue damage in the xylem of some branch samples used for another study we conducted on the same trees, which may indicate that branches were affected by frost events prior to the period when wood formation had started in the lower stem parts. Conifer species can still maintain their cambial activity in February and March with low *minimum* temperature (<0 °C; see Begum *et al.*, 2010). Moreover, it was shown that the temperature of cambial activity (i.e. production of new cells) for conifer species varied between 10 and 14 °C for *maximum* air temperature (Rossi *et al*., 2008). In our study site, the conifer species had already experienced high maximum temperatures of between 10 and 14 °C in January or February. It is very likely that the species had already started cambial activity and were going to be affected by frost days in March and April. Hence, spring frosts may reduce cambial activity and growth. In addition, spring forst may damage buds and delay the start of the growing season for leaf-out (Zohner *et al.*, 2020), or probably cause freezing-induced cavitation of newly formed tracheids, thus impairing water transport (Pittermann and Sperry, 2003), or leading to stomatal closure or desiccation and tissue loss in the leaves (Davis *et al.*, 1999; Mayr *et al.*, 2006), and resulting in a decline in photosynthetic activity and carbon gain (Augspurger, 2011; Vitra *et al.*, 2017). Although such mechanisms have been poorly studied, this suggests that species differences in the timing of stem growth and leaf or cambial phenology may be important for explaining species differences in sensitivity to spring frost.

### Are species with a high growth potential also more sensitive to climatic variation?

We hypothesized that species with a high growth potential would also be more sensitive to climatic variation. It was assumed that traits associated with high inherent growth rates, such as large cells with thin cell walls that facilitate high metabolic activity, would lead to a higher sensitivity to extreme climate events such as spring frost or summer drought. However, we only observed a weak pattern that contrasted with the expected positive relationship, since stem diameter growth and growth sensitivity to summer drought were negatively – and not positively – associated (*r* = −0.40, *P* = 0.09) (Fig. 3, Table 3). In particular, *Abies grandis* combined a large growth potential with high tolerance to climate extremes, as shown by the low impact of spring frost and summer drought on stem growth of this species (Table 2). Among all investigated species, *Abies grandis* had the second-largest tracheid diameter (14.0 ± 0.8 µm) (Y. Song *et al*., unpubl. data), which allows the species to have a high hydraulic conductivity, high transpiration rates and photosynthetic rates, and thus a large wood production (Chave *et al.*, 2009). Possible detrimental effects of climate extremes could be counterbalanced by the high stomatal conductance even during drought (Puritch, 1973) or the potentially deep roots that can ensure water uptake from deeper soil layers under dry conditions (Foiles *et al*., 1990; Xu *et al.*, 1997). Also, the relatively mild maritime climate in our study site creates favourable growing conditions during large parts of the growing season and may have resulted in a weaker relationship between growth potential and climate sensitivity for our study species. Overall, our study implies that, for a given site, forest managers have the option to select and plant conifer species that combine a high growth rate with a high tolerance to summer droughts. This is an important prerequisite for designing climate-smart forests for the future (Nabuurs *et al.*, 2018) that combine high productivity with large carbon storage potential and strong drought resilience.

### Is growth potential of conifers phylogenetically conserved?

Since conifers radiated in recent evolutionary times into different habitats (Zanne *et al.*, 2014), we hypothesized that the growth potential and climate sensitivity of the growth of conifer species are phylogenetically weakly conserved. Although weak, we nevertheless found some indications for phylogenetical control of stem growth (Table 4, Fig. 2) and that, for example, *Abies* and *Picea* species grow faster than *Taxus* species. In our common garden experiment established under maritime climate conditions, *Abies grandis* was the fastest-growing species, with an average radial stem growth rate of 0.61 cm yr^–1^, while the two most slow-growing species were *Taxus baccata* (0.20 cm yr^–1^) and *Taxus cuspidata* (0.19 cm yr^–1^) (Fig. 2). *Abies grandis* can grow fast because it has fast-growth traits, such as relatively wide tracheids, high specific leaf area and high photosynthetic rates, compared to other species (Y. Song *et al*., unpubl. results). *Taxus* has very dense and heavy wood with narrow tracheids, traits that tend to reduce growth rates (Thomas and Polwart, 2003). Overall, species differences in productivity are only partially phylogenetically controlled.

### Is growth sensitivity to climate of conifers phylogenetically conserved?

Of the five seasonal potentially growth-limiting climate conditions, only the growth sensitivity to spring frost and previous summer water availability (summer SPEI) were found to be phylogenetically controlled (Table 4). *Larix kaempferi* and several *Picea* species, in particular, decreased stem growth in response to spring frost (Fig. 2) and each species might be sensitive for a different reason. First, *Larix kaempferi*, a winter deciduous species, normally starts to open buds in early May and it takes 3 weeks to fully extend leaves (Hirano *et al.*, 2003; Nakagawa, 2021). The late spring frosts between March and April in our study area may damage larch buds, and consequently limit leaf expansion and reduce growth. The probably earlier timing of leaf flush for larch due to global warming may thus maintain such spring frost risks for this species. Second, *Picea omorika* showed the second strongest negative growth response to spring frosts (Fig. 2, Table 2), while it has been considered tolerant (Dallimore, 1937). Possibly, the contrast between its natural distribution restricted to the banks of the river Drina in south-eastern Europe and the water-drained sandy soils affects the phenology of the species and causes high risks of late spring frosts (Dallimore, 1937; Schmidt, 2003). Third, for *Picea abies*, the low frost resistance may be related to a reduced solute concentration of the cell sap (i.e. much less negative osmotic potential at full saturation), reducing the osmotic potential and increasing freezing and damaging ice crystal formation in stem cells (Santarius, 1973; Neuner and Beikircher, 2010). Fourth, for *Taxus* species, it is known that a high content of leaf mucilage can cause strong water-binding and prevent frost damage (Distelbarth and Kull, 1985). Overall, our results imply that early production of leaves in combination with high sensitivity to winter frost may cause stronger stem growth reductions for *Larix*, *Picea* and *Pseudotsuga menziesii* compared to, for example, *Taxus* and *Abies.*

Remarkably, growth sensitivity to previous summer SPEI seems to be phylogenetically controlled, whereas growth sensitivity to current summer SPEI is not phylogenetically controlled (Table 4). *Larix* and *Pseudotsuga* belong to the same phylogenetic clade, and both species significantly increased their stem growth in response to previous summer SPEI, possibly as a result of the strategy to increase carbohydrate storage for the next year or the formation of more leaf buds for the following year. In contrast, *Tsuga* species (Fig. 3) – and particularly *Tsuga diversifolia* (Table 2) – reduced their stem growth in response to previous summer rain, for which we do not have a clear explanation.

## IMPLICATIONS FOR FOREST MANAGEMENT UNDER CLIMATE CHANGE

Climate change scenarios predict a warmer, drier and more variable climate for Europe (e.g. Ballester *et al.*, 2010). Warmer spring conditions can induce an earlier start of the growing season, which advances stem and leaf phenology, with increased risks of damage by late spring frosts (Vitasse *et al.*, 2018). Given that all 19 conifer species in our common garden experiment reduced stem growth with reduced water availability during summer and 37 % of species reduced stem growth with more spring frosts, a warmer future climate will probably reduce the productivity of conifer species and forests. This not only applies to the maritime climate of our north-west European study site, but also to many other regions in the northern hemisphere (Hogg *et al.*, 2002; Montwé *et al.*, 2018). Our study nevertheless implies that forest managers can design relatively climate-smart forests by favouring those species that combine a high growth rate with a positive response to winter warming (e.g. *Tsuga heterophylla*), or low sensitivity to spring frost (e.g. *Abies grandis*) or summer drought (e.g. *Abies grandis*) for temperate maritime climate conditions. While such species choices probably depend on local specific site conditions, comparative information on stem growth responses to climate can help create productive and resilient forests for a warmer and drier future.

## SUPPLEMENTARY DATA

Supplementary data are available online at https://academic.oup.com/aob and consist of the following. Fig. S1: Climate change of the De Bilt weather station, the Netherlands. Fig. S2: The plots for residuals in the mixed model for growth potential. Fig. S3: The plots for residuals in the mixed model for growth sensitivity. Fig. S4: Relationships between cumulative growth and ages. Fig. S5: Alternative proxies of stem growth potential for 19 conifer species. Table S1: Summary statistics related to cross-dating are provided for 19 conifer species. Table S2: Species-specific *R*^2^ was calculated from the square of correlations between the predicted tree ring index and the observed tree ring index.

mcab090_suppl_Supplementary_Material

## References

[CIT0001] Adams DC. 2014. A generalized K statistic for estimating phylogenetic signal from shape and other high-dimensional multivariate data. Systematic Biology 63: 685–697. 24789073 10.1093/sysbio/syu030

[CIT0001a] Adams HD, Zeppel MJ, Anderegg WR, et al. 2017. A multi-species synthesis of physiological mechanisms in drought-induced tree mortality. Nature Ecology and Evolution 1: 1285–1291. 29046541 10.1038/s41559-017-0248-x

[CIT0002] Augspurger CK. 2011. Frost damage and its cascading negative effects on Aesculusglabra. Plant Ecology 212: 1193–1203.

[CIT0003] Babst F, Poulter B, Trouet V, et al. 2013. Site-and species-specific responses of forest growth to climate across the European continent. Global Ecology and Biogeography 22: 706–717.

[CIT0004] Ballester J, Rodó X, Giorgi F. 2010. Future changes in Central Europe heat waves expected to mostly follow summer mean warming. Climate Dynamics 35: 1191–1205.

[CIT0002a] Beguería S, Vicente-Serrano SM, Beguería MS. 2017. Package ‘SPEI’. https://cran.r-project.org/web/packages/SPEI/index.html

[CIT0006] Begum S, Nakaba S, Oribe Y, Kubo T, Funada R. 2010. Cambial sensitivity to rising temperatures by natural condition and artificial heating from late winter to early spring in the evergreen conifer *Cryptomeria japonica*. Trees 24: 43–52.

[CIT0007] Bhuyan U, Zang C, Menzel A. 2017. Different responses of multispecies tree ring growth to various drought indices across Europe. Dendrochronologia 44: 1–8.

[CIT0008] Biondi F. 1997. Evolutionary and moving response functions in dendroclimatology. Dendrochronologia 15: 139–150.

[CIT0009] Biondi F, Waikul K. 2004. DENDROCLIM2002: A C++ program for statistical calibration of climate signals in tree-ring chronologies. Computers & Geosciences 30: 303–311.

[CIT0010] Blackwell P, Rennolls K, Coutts M. 1990. A root anchorage model for shallowly rooted Sitka spruce. Forestry: An International Journal of Forest Research 63: 73–91.

[CIT0011] Blomberg SP, Garland T Jr, Ives AR. 2003. Testing for phylogenetic signal in comparative data: behavioral traits are more labile. Evolution; International Journal of Organic Evolution 57: 717–745. 12778543 10.1111/j.0014-3820.2003.tb00285.x

[CIT0012] Bolte A, Villanueva I. 2006. Interspecific competition impacts on the morphology and distribution of fine roots in European beech (*Fagus sylvatica* L.) and Norway spruce (*Picea abies* (L.) Karst.). European Journal of Forest Research 125: 15–26.

[CIT0003a] Bottero A, Forrester DI, Cailleret M, et al. 2021. Growth resistance and resilience of mixed silver fir and Norway spruce forests in central Europe: contrasting responses to mild and severe droughts. Global Change Biology 1–17. 34166562 10.1111/gcb.15737 PMC8453522

[CIT0013] Bunn AG. 2008. A dendrochronology program library in R (dplR). Dendrochronologia 26: 115–124.

[CIT0014] CaraDonna PJ, Inouye DW. 2015. Phenological responses to climate change do not exhibit phylogenetic signal in a subalpine plant community. Ecology 96: 355–361. 26240857 10.1890/14-1536.1

[CIT0015] Charney ND, Babst F, Poulter B, et al. 2016. Observed forest sensitivity to climate implies large changes in 21st century North American forest growth. Ecology Letters 19: 1119–1128. 27434040 10.1111/ele.12650

[CIT0016] Chave J, Coomes D, Jansen S, Lewis SL, Swenson NG, Zanne AE. 2009. Towards a worldwide wood economics spectrum. Ecology Letters 12: 351–366. 19243406 10.1111/j.1461-0248.2009.01285.x

[CIT0004a] Choat B, Brodribb TJ, Brodersen CR, Duursma RA, Lopez R, Medlyn BE. 2018. Triggers of tree mortality under drought. Nature 558: 531–539. 29950621 10.1038/s41586-018-0240-x

[CIT0017] Chudnovskii A. 1949. Zamorozki (Light Frosts). Leningrad: Gidrometeoizdat.

[CIT0018] Ciais P, Reichstein M, Viovy N, et al. 2005. Europe-wide reduction in primary productivity caused by the heat and drought in 2003. Nature 437: 529–533. 16177786 10.1038/nature03972

[CIT0005a] Cook ER, Briffa K, Shiyatov S, Mazepa V. 1990. Tree-ring standardization and growth-trend estimation. In: Cook ER, Kairiukstis LA, eds. Methods of dendrochronology: applications in the environmental sciences. Dordrecht, The Netherlands: Kluwer Academic Publishers, 104–123.

[CIT0020] Cook ER, Briffa KR, Meko DM, Graybill DA, Funkhouser G. 1995. The’segment length curse’in long tree-ring chronology development for palaeoclimatic studies. The Holocene 5: 229–237.

[CIT0021] Cornelissen JH, Sass-Klaassen U, Poorter L, et al. 2012. Controls on coarse wood decay in temperate tree species: birth of the LOGLIFE experiment. Ambio 41(Suppl 3): 231–245. 22864697 10.1007/s13280-012-0304-3 PMC3535053

[CIT0022] Cuny HE, Rathgeber CB. 2016. Xylogenesis: coniferous trees of temperate forests are listening to the climate tale during the growing season but only remember the last words! Plant Physiology 171: 306–317. 27208048 10.1104/pp.16.00037 PMC4854703

[CIT0023] Cuny HE, Rathgeber CB, Lebourgeois F, Fortin M, Fournier M. 2012. Life strategies in intra-annual dynamics of wood formation: example of three conifer species in a temperate forest in north-east France. Tree Physiology 32: 612–625. 22543476 10.1093/treephys/tps039

[CIT0024] D’Orangeville L, Duchesne L, Houle D, Kneeshaw D, Côté B, Pederson N. 2016. Northeastern North America as a potential refugium for boreal forests in a warming climate. Science (New York, N.Y.) 352: 1452–1455. 10.1126/science.aaf4951 27313044

[CIT0025] D’Orangeville L, Maxwell J, Kneeshaw D, et al. 2018. Drought timing and local climate determine the sensitivity of eastern temperate forests to drought. Global Change Biology 24: 2339–2351. 29460369 10.1111/gcb.14096

[CIT0026] Dallimore W. 1937. The more recently introduced conifers and their value for planting in the British Isles. Forestry: An International Journal of Forest Research 11: 1–5.

[CIT0027] Davis SD, Sperry JS, Hacke UG. 1999. The relationship between xylem conduit diameter and cavitation caused by freezing. American Journal of Botany 86: 1367–1372. 10523278

[CIT0028] DeSoto L, Varino F, Andrade JP, et al. 2014. Different growth sensitivity to climate of the conifer *Juniperus thurifera* on both sides of the Mediterranean Sea. International Journal of Biometeorology 58: 2095–2109. 24659114 10.1007/s00484-014-0811-y

[CIT0029] Dietrich L, Delzon S, Hoch G, Kahmen A. 2019. No role for xylem embolism or carbohydrate shortage in temperate trees during the severe 2015 drought. Journal of Ecology 107: 334–349.

[CIT0030] Distelbarth H, Kull U. 1985. Physiological investigations of leaf mucilages II. The Mucilage of *Taxus baccata* L. and of *Thuja occidentale* L. Israel Journal of Plant Sciences 34: 113–128.

[CIT0031] Dy G, Payette S. 2007. Frost hollows of the boreal forest as extreme environments for black spruce tree growth. Canadian Journal of Forest Research 37: 492–504.

[CIT0032] Eysteinsson T, Karlman L, Fries A, Martinsson O, Skulason B. 2009. Variation in spring and autumn frost tolerance among provenances of Russian larches (*Larix* Mill.). Scandinavian Journal of Forest Research 24: 100–110.

[CIT0033] Farjon A, Filer D. 2013. An atlas of the world’s conifers: an analysis of their distribution, biogeography, diversity and conservation status. Leiden: Brill.

[CIT0006a] Foiles MW, Graham RT, Olson DF, Jr. 1990. Abies grandis (Dougl. ex. D. Don) Lindl. Grand Fir. In: Burns RM, Honkala BH, tech. coords. Silvics of North America: 1. Conifers. Agriculture Handbook 654. Washington, DC: USDA Forest Service, 80–96.

[CIT0035] Fritts H. 1976. Tree rings and climate. London: Academic Press.

[CIT0036] Fry D, Phillips I. 1977. Photosynthesis of conifers in relation to annual growth cycles and dry matter production: II. Seasonal photosynthetic capacity and mesophyll ultrastructure in *Abies grandis*, *Picea sitchensis*, *Tsuga heterophylla* and *Larix leptolepis* growing in SW England. Physiologia Plantarum 40: 300–306.

[CIT0037] Gärtner H, Nievergelt D. 2010. The core-microtome: a new tool for surface preparation on cores and time series analysis of varying cell parameters. Dendrochronologia 28: 85–92.

[CIT0038] Gazol A, Camarero JJ, Vicente-Serrano SM, et al. 2018. Forest resilience to drought varies across biomes. Global Change Biology 24: 2143–2158. 29488293 10.1111/gcb.14082

[CIT0039] Gazol A, Camarero JJ, Colangelo M, de Luis M, del Castillo EM, Serra-Maluquer X. 2019. Summer drought and spring frost, but not their interaction, constrain European beech and silver fir growth in their southern distribution limits. Agricultural and Forest Meteorology 278: 107695.

[CIT0040] Gould IJ, Quinton JN, Weigelt A, De Deyn GB, Bardgett RD. 2016. Plant diversity and root traits benefit physical properties key to soil function in grasslands. Ecology Letters 19: 1140–1149. 27459206 10.1111/ele.12652 PMC4988498

[CIT0041] Gurskaya M. 2014. Temperature conditions of the formation of frost damages in conifer trees in the high latitudes of Western Siberia. Biology Bulletin 41: 187–196. 25735171

[CIT0042] Hartmann H. 2011. Will a 385 million year-struggle for light become a struggle for water and for carbon?–How trees may cope with more frequent climate change-type drought events. Global Change Biology 17: 642–655.

[CIT0043] Harvey JE, Smiljanić M, Scharnweber T, et al. 2020. Tree growth influenced by warming winter climate and summer moisture availability in northern temperate forests. Global Change Biology 26: 2505–2518. 10.1111/gcb.14966 31860143

[CIT0044] Hirano T, Hirata R, Fujinuma Y, et al 2003. CO_2_ and water vapor exchange of a larch forest in northern Japan. Tellus B: Chemical and Physical Meteorology 55: 244–257.

[CIT0045] Hogg E, Brandt JP, Kochtubajda B. 2002. Growth and dieback of aspen forests in northwestern Alberta, Canada, in relation to climate and insects. Canadian Journal of Forest Research 32: 823–832.

[CIT0046] Huang B. 2000. Role of root morphological and physiological characteristics in drought resistance of plants. Plant–Environment Interactions. New York: Marcel Dekker Inc., 39–64.

[CIT0047] Huang J, Tardif JC, Bergeron Y, Denneler B, Berninger F, Girardin MP. 2010. Radial growth response of four dominant boreal tree species to climate along a latitudinal gradient in the eastern Canadian boreal forest. Global Change Biology 16: 711–731.

[CIT0048] Huang W, Fonti P, Larsen JB, et al. 2017. Projecting tree-growth responses into future climate: a study case from a Danish-wide common garden. Agricultural and Forest Meteorology 247: 240–251.

[CIT0049] Huang M, Wang X, Keenan TF, Piao S. 2018. Drought timing influences the legacy of tree growth recovery. Global Change Biology 24: 3546–3559. 29729065 10.1111/gcb.14294

[CIT0050] Inouye DW. 2000. The ecological and evolutionary significance of frost in the context of climate change. Ecology Letters 3: 457–463.

[CIT0051] Julio Camarero J, Gazol A, Sangüesa-Barreda G, et al. 2018. Forest growth responses to drought at short- and long-term scales in spain: squeezing the stress memory from tree rings. Frontiers in Ecology and Evolution 6.

[CIT0052] Kembel SW, Cowan PD, Helmus MR, et al. 2010. Picante: R tools for integrating phylogenies and ecology. Bioinformatics (Oxford, England) 26: 1463–1464. 10.1093/bioinformatics/btq166 20395285

[CIT0053] Klesse S, Babst F, Lienert S, et al. 2018. A combined tree ring and vegetation model assessment of European forest growth sensitivity to interannual climate variability. Global Biogeochemical Cycles 32: 1226–1240.

[CIT0054] Kurz WA, Dymond CC, Stinson G, et al. 2008. Mountain pine beetle and forest carbon feedback to climate change. Nature 452: 987–990. 18432244 10.1038/nature06777

[CIT0055] Larcher W. 2000. Temperature stress and survival ability of Mediterranean sclerophyllous plants. Plant Biosystems 134: 279–295.

[CIT0056] Larsen KS, Ibrom A, Jonasson S, Michelsen A, Beier C. 2007. Significance of cold-season respiration and photosynthesis in a subarctic heath ecosystem in Northern Sweden. Global Change Biology 13: 1498–1508.

[CIT0057] Li W-F, Ding Q, Cui K-M, He X-Q. 2013. Cambium reactivation independent of bud unfolding involves de novo IAA biosynthesis in cambium regions in *Populus tomentosa* Carr. Acta Physiologiae Plantarum 35: 1827–1836.

[CIT0058] Markesteijn L, Poorter L. 2009. Seedling root morphology and biomass allocation of 62 tropical tree species in relation to drought-and shade-tolerance. Journal of Ecology 97: 311–325.

[CIT0059] Mayr S, Hacke U, Schmid P, Schwienbacher F, Gruber A. 2006. Frost drought in conifers at the alpine timberline: xylem dysfunction and adaptations. Ecology 87: 3175–3185. 17249241 10.1890/0012-9658(2006)87[3175:fdicat]2.0.co;2

[CIT0060] McDowell N, Pockman WT, Allen CD, et al. 2008. Mechanisms of plant survival and mortality during drought: why do some plants survive while others succumb to drought? The New Phytologist 178: 719–739. 18422905 10.1111/j.1469-8137.2008.02436.x

[CIT0061] McLane SC, LeMay VM, Aitken SN. 2011. Modeling lodgepole pine radial growth relative to climate and genetics using universal growth-trend response functions. Ecological Applications 21: 776–788. 21639044 10.1890/10-0131.1

[CIT0062] Montwé D, Isaac-Renton M, Hamann A, Spiecker H. 2018. Cold adaptation recorded in tree rings highlights risks associated with climate change and assisted migration. Nature Communications 9: 1574. 10.1038/s41467-018-04039-5 PMC5913219 29686289

[CIT0063] Nabuurs G-J, Verkerk PJ, Schelhaas M, González-Olabarria J, Trasobares A, Cienciala E. 2018. Climate-Smart Forestry: mitigation implact in three European regions. Sarjanr, Finland: European Forest Institute.

[CIT0007a] Nakagawa M. 2021. Effect of Japanese larch arable land windbreaks on wind damage reduction in the early spring cultivation season: a case study in Kamioribe District, Shihoro Town, Eastern Hokkaido. Journal of Forest Planning 27: 1–9.

[CIT0065] Neuner G, Beikircher B. 2010. Critically reduced frost resistance of *Picea abies* during sprouting could be linked to cytological changes. Protoplasma 243: 145–152. 19533300 10.1007/s00709-009-0052-9

[CIT0066] Oberhuber W, Gruber A, Lethaus G, Winkler A, Wieser G. 2017. Stem girdling indicates prioritized carbon allocation to the root system at the expense of radial stem growth in Norway spruce under drought conditions. Environmental and Experimental Botany 138: 109–118. 28392608 10.1016/j.envexpbot.2017.03.004 PMC5381714

[CIT0067] Pan Y, Birdsey RA, Fang J, et al. 2011. A large and persistent carbon sink in the world’s forests. Science (New York, N.Y.) 333: 988–993. 10.1126/science.1201609 21764754

[CIT0068] Park C-W, Ko S, Yoon TK, et al. 2012. Differences in soil aggregate, microbial biomass carbon concentration, and soil carbon between *Pinus rigida* and *Larix kaempferi* plantations in Yangpyeong, central Korea. Forest Science and Technology 8: 38–46.

[CIT0008a] Pinheiro J, Bates D, DebRoy S, et al. 2017. Package ‘nlme’: Linear and nonlinear mixed effects models, version 3-1. https://CRAN.R-project.org/package=nlme.

[CIT0070] Piper FI, Fajardo A, Hoch G. 2017. Single-provenance mature conifers show higher non-structural carbohydrate storage and reduced growth in a drier location. Tree Physiology 37: 1001–1010. 28549182 10.1093/treephys/tpx061

[CIT0009a] Pittermann J, Sperry J. 2003. Tracheid diameter is the key trait determining the extent of freezing-induced embolism in conifers. Tree Physiology 23: 907–914. 14532014 10.1093/treephys/23.13.907

[CIT0071] Pittermann J, Sperry JS, Wheeler JK, Hacke UG, Sikkema EH. 2006. Mechanical reinforcement of tracheids compromises the hydraulic efficiency of conifer xylem. Plant, Cell & Environment 29: 1618–1628. 10.1111/j.1365-3040.2006.01539.x 16898022

[CIT0072] Puchi PF, Castagneri D, Rossi S, Carrer M. 2020. Wood anatomical traits in black spruce reveal latent water constraints on the boreal forest. Global Change Biology 26: 1767–1777. 31692158 10.1111/gcb.14906

[CIT0073] Puritch GS. 1973. Effect of water stress on photosynthesis, respiration, and transpiration of four *Abies* species. Canadian Journal of Forest Research 3: 293–298.

[CIT0010a] Rossi S, Deslauriers A, Griçar J, et al. 2008. Critical temperatures for xylogenesis in conifers of cold climates. Global Ecology and Biogeography 17: 696–707.

[CIT0074] R Core Team. 2019. *R: A language and environment for statistical computing*. Vienna, Austria: R Foundation for Statistical Computing. Retrieved from https://www.R-project.org/

[CIT0075] Revell LJ. 2012. phytools: an R package for phylogenetic comparative biology (and other things). Methods in Ecology and Evolution 3: 217–223.

[CIT0076] Sala A, Piper F, Hoch G. 2010. Physiological mechanisms of drought-induced tree mortality are far from being resolved. The New Phytologist 186: 274–281. 20409184 10.1111/j.1469-8137.2009.03167.x

[CIT0077] Santarius KA. 1973. The protective effect of sugars on chloroplast membranes during temperature and water stress and its relationship to frost, desiccation and heat resistance. Planta 113: 105–114. 24468903 10.1007/BF00388196

[CIT0078] Saunders MR, Wagner RG. 2008. Height-diameter models with random coefficients and site variables for tree species of Central Maine. Annals of Forest Science 65: 1–10.

[CIT0079] Schmid I, Kazda M. 2001. Vertical distribution and radial growth of coarse roots in pure and mixed stands of *Fagus sylvatica* and *Picea abies*. Canadian Journal of Forest Research 31: 539–548.

[CIT0011a] Schmidt PA. 2003. The diversity, phytogeography and ecology of spruces (Picea: Pinaceae) in Eurasia. Acta Hortic 615:189–201. doi:10.17660/ActaHortic.2003.615.18.

[CIT0081] Shestakova TA, Gutiérrez E, Kirdyanov AV, et al. 2016. Forests synchronize their growth in contrasting Eurasian regions in response to climate warming. Proceedings of the National Academy of Sciences of the United States of America 113: 662–667. 26729860 10.1073/pnas.1514717113 PMC4725483

[CIT0082] Sperry JS, Hacke UG, Pittermann J. 2006. Size and function in conifer tracheids and angiosperm vessels. American Journal of Botany 93: 1490–1500. 21642096 10.3732/ajb.93.10.1490

[CIT0083] Sterck FJ, Martínez-Vilalta J, Mencuccini M, et al. 2012. Understanding trait interactions and their impacts on growth in Scots pine branches across Europe. Functional Ecology 26: 541–549.

[CIT0084] Takahashi K, Obata Y. 2014. Growth, allometry and shade tolerance of understory saplings of four subalpine conifers in central Japan. Journal of Plant Research 127: 329–338. 24310614 10.1007/s10265-013-0610-2

[CIT0085] Thomas P, Polwart A. 2003. *Taxus baccata* L. Journal of Ecology 91: 489–524.

[CIT0086] Thornthwaite CW. 1948. An approach toward a rational classification of climate. Geographical Review 38: 55–94.

[CIT0087] TNO-NITG DINOloket. 2020. www.cinoloket.nl. Accessed January 2020.

[CIT0088] Vicente-Serrano SM, Beguería S, López-Moreno JI. 2010. A multiscalar drought index sensitive to global warming: the standardized precipitation evapotranspiration index. Journal of climate 23: 1696–1718.

[CIT0089] Vitali V, Büntgen U, Bauhus J. 2017. Silver fir and Douglas fir are more tolerant to extreme droughts than Norway spruce in south-western Germany. Global Change Biology 23: 5108–5119. 28556403 10.1111/gcb.13774

[CIT0090] Vitasse Y, Signarbieux C, Fu YH. 2018. Global warming leads to more uniform spring phenology across elevations. Proceedings of the National Academy of Sciences of the United States of America 115: 1004–1008. 29279381 10.1073/pnas.1717342115 PMC5798366

[CIT0091] Vitasse Y, Bottero A, Cailleret M, et al. 2019. Contrasting resistance and resilience to extreme drought and late spring frost in five major European tree species. Global Change Biology 25: 3781–3792. 31436853 10.1111/gcb.14803

[CIT0092] Vitra A, Lenz A, Vitasse Y. 2017. Frost hardening and dehardening potential in temperate trees from winter to budburst. The New Phytologist 216: 113–123. 28737248 10.1111/nph.14698

[CIT0093] van der Wal A, Klein Gunnewiek PJ, Cornelissen JHC, Crowther TW, de Boer W. 2016. Patterns of natural fungal community assembly during initial decay of coniferous and broadleaf tree logs. Ecosphere 7: e01393.

[CIT0094] Walker AP, De Kauwe MG, Bastos A, et al. 2021. Integrating the evidence for a terrestrial carbon sink caused by increasing atmospheric CO_2_. The New Phytologist 229: 2413–2445. 32789857 10.1111/nph.16866

[CIT0095] Weemstra M, Eilmann B, Sass-Klaassen UG, Sterck FJ. 2013. Summer droughts limit tree growth across 10 temperate species on a productive forest site. Forest Ecology and Management 306: 142–149.

[CIT0096] Williams AP, Michaelsen J, Leavitt SW, Still CJ. 2010. Using tree rings to predict the response of tree growth to climate change in the continental United States during the twenty-first century. Earth Interactions 14: 1–20.

[CIT0097] Williams CM, Henry HA, Sinclair BJ. 2015. Cold truths: how winter drives responses of terrestrial organisms to climate change. Biological Reviews of the Cambridge Philosophical Society 90: 214–235. 24720862 10.1111/brv.12105

[CIT0098] Willinge Gratama-Oudemans JJ. 1992. The arboretum of Schovenhorst, Putten, in the Netherlands. Arboricultural Journal 16: 197–205.

[CIT0099] Xu Y-J, Röhrig E, Fölster H. 1997. Reaction of root systems of grand fir (*Abies grandis* Lindl.) and Norway spruce (*Picea abies* Karst.) to seasonal waterlogging. Forest Ecology and Management 93: 9–19.

[CIT0100] Zanne AE, Tank DC, Cornwell WK, et al. 2014. Three keys to the radiation of angiosperms into freezing environments. Nature 506: 89–92. 24362564 10.1038/nature12872

[CIT0101] Zohner CM, Mo L, Renner SS, et al. 2020. Late-spring frost risk between 1959 and 2017 decreased in North America but increased in Europe and Asia. Proceedings of the National Academy of Sciences of the United States of America 117: 12192–12200. 32393624 10.1073/pnas.1920816117 PMC7275740

