# Supplementary material for: Growth of 19 conifer species is highly sensitive to winter warming, spring frost and summer drought
Source: Ann Bot. 2021 Jul 30;128(5):545–57. doi: 10.1093/aob/mcab090 (PMC8422889; doi:10.1093/aob/mcab090)
Supplement: mcab090_suppl_Supplementary_Material [file mcab090_suppl_supplementary_material.docx]

## Supporting Information

Article title: **Growth of 19 conifer species is highly sensitive to winter warming, spring frost and summer drought**

Authors: Yanjun Song, Ute Sass-Klaassen, Frank Sterck, Leo Goudzwaard, Linar Akhmetzyanov, Lourens Poorter

The following Supporting Information is available for this article:

**Fig. S1** Climate change of the De Bilt weather station, the Netherlands: (a), monthly mean precipitation (bars) and temperature (line) over the common period 1974–2017; (b), annual mean temperature over the period from 1901 to 2018; (c) annual aggregate frost days over the period from 1901 to 2018. Regression lines and 95% confidence intervals (grey), coefficients of determination (R^2^) and *P*-value are shown.

**Fig. S2** The plots for residuals in the mixed model for growth potential for a) a Q-Q (quantile-quantile) plot, b) a residual plot, c) a frequency diagram for the residuals.

**Fig. S3** The plots for residuals in the mixed model for growth sensitivity for a) a Q-Q (quantile-quantile)) plot, b) a residual plot, c) a frequency diagram for the residuals.

**Fig.** **S4** Relationships between cumulative growth and ages for a) cumulative ring width and the whole period, b) cumulative ring width and the first twenty years, c) cumulative basal area and the whole period, d) cumulative basal area and the first twenty years.

**Fig. S5** Alternative proxies of stem growth potential for 19 conifer species for a) stem diameter growth (in cm) based on first twenty years, b) stem area growth (in cm^2^/cm) over the period between 1911-2018 (*C. lawsoniana*) and 1981-2018 (*P. armandii*), c) stem mass growth (in kg yr^-1^ m^-1^) calculated from stem area growth and wood density. Error bars (± standard error of the mean value) are shown.

**Table S1** Summary statistics related to cross-dating are provided for 19 conifer species.

**Table S2** Species-specific *R^2^* was calculated from the square of correlations between the predicted tree ring index (based on the multiple regression in Table 2) and the observed tree ring index. *R^2^ind* indicates species-specific *R^2^* based on the individual level and *R^2^sp* indicates species-specific *R^2^* based on mean chronology (i.e., species) level.

**Figure S1**


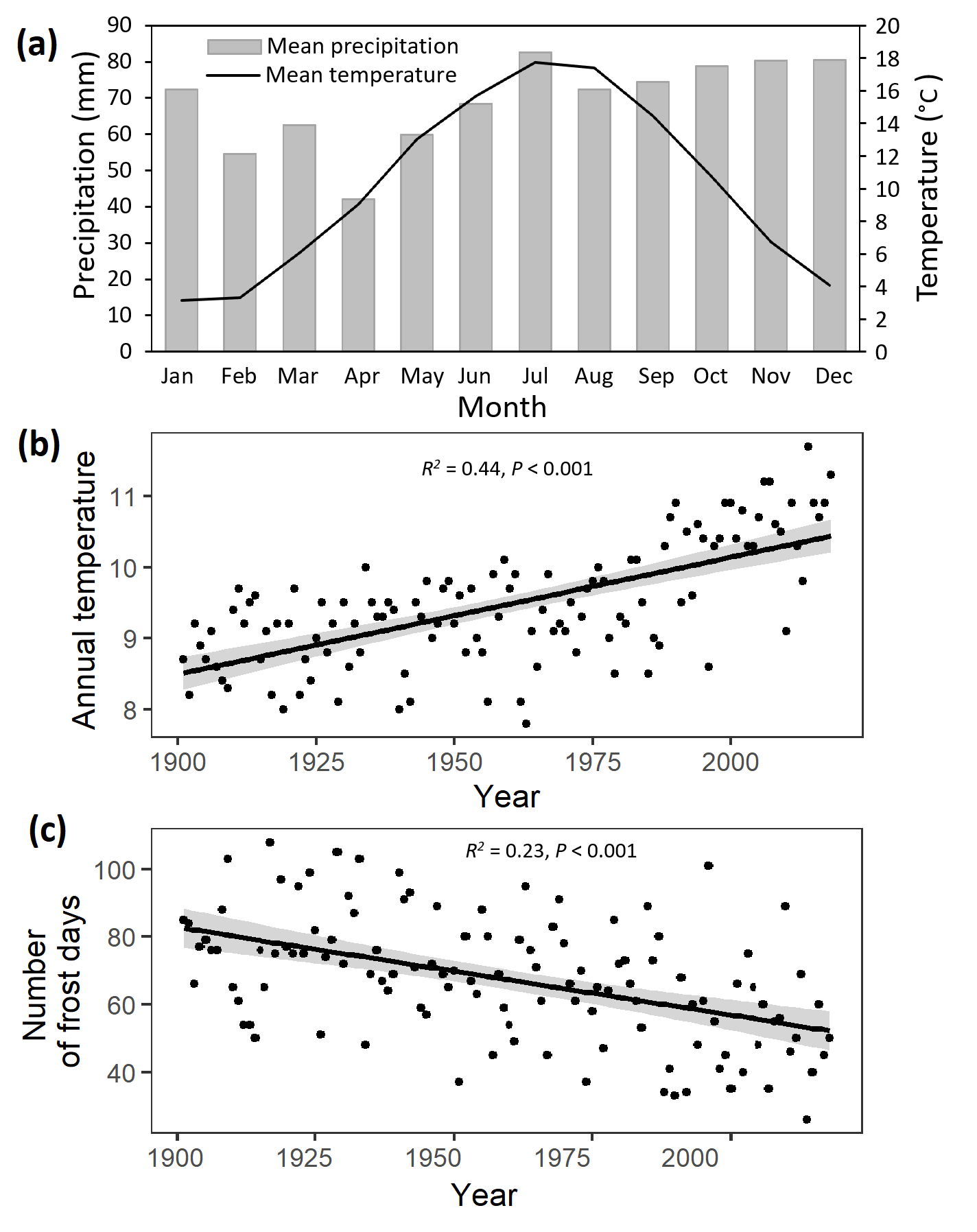


**Figure S2**


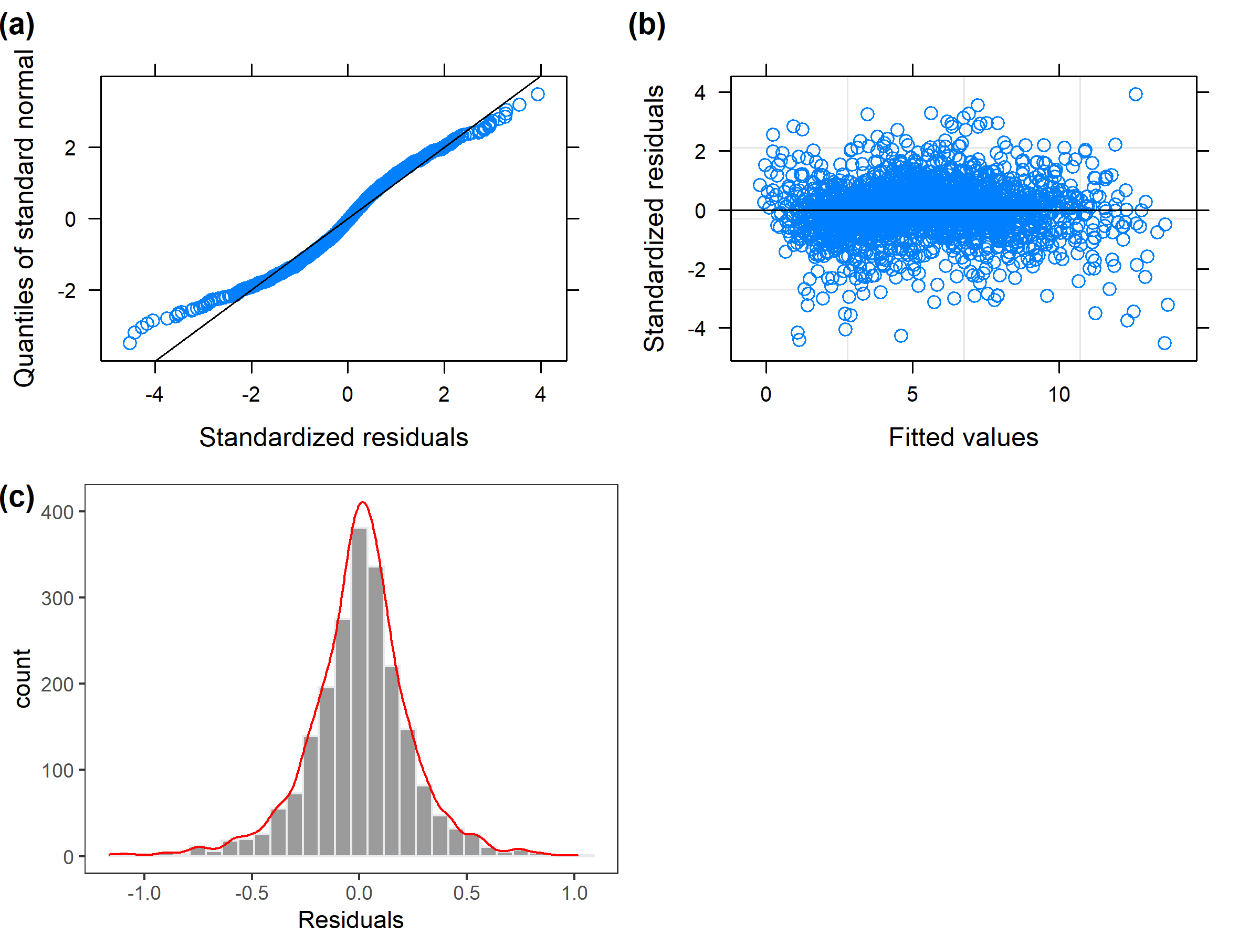


**Figure S3**


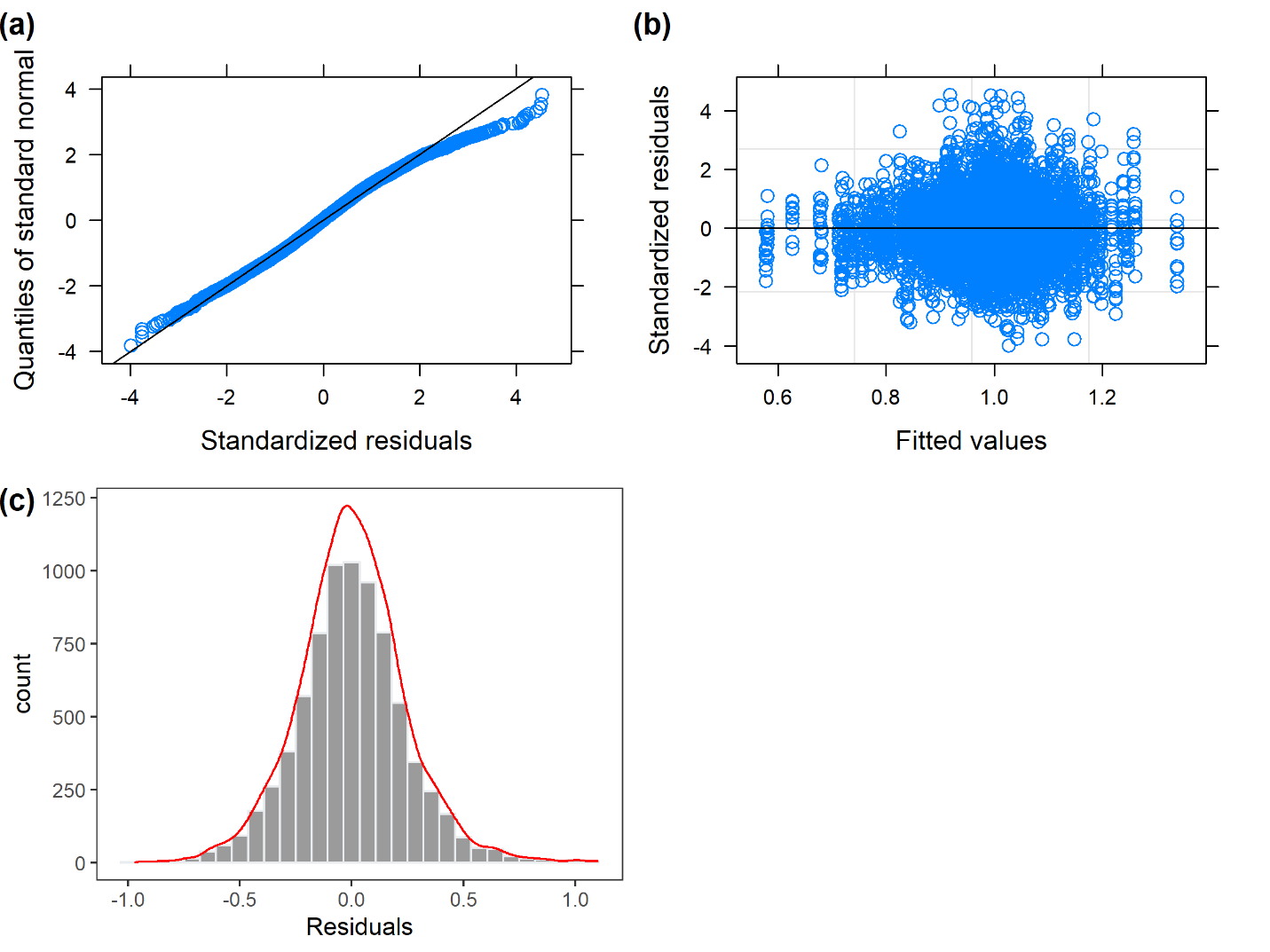
**Figure S4**


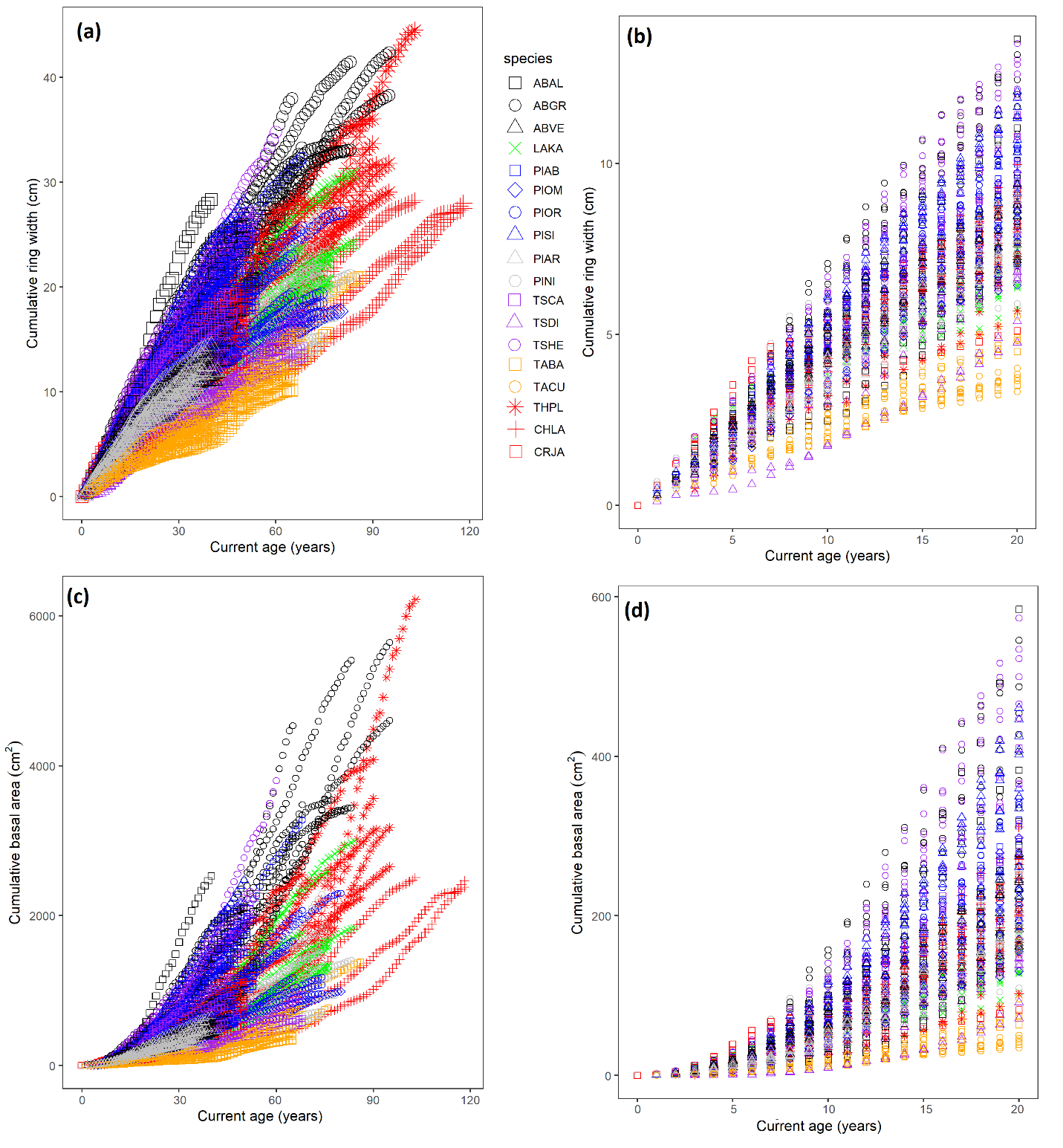


**Figure S5**


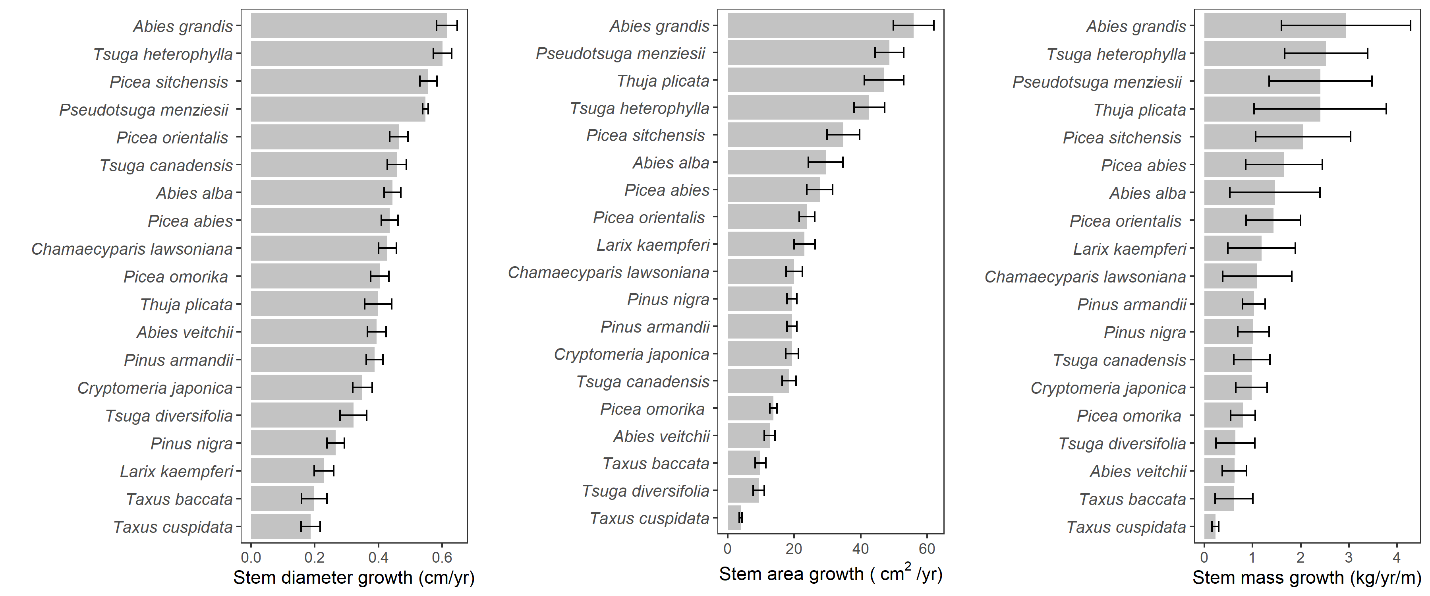


**Table S1** Summary statistics related to cross-dating are provided for 19 conifer species**.**

| Species | Period considered | Mean ring width (sd) (mm) | Rbar | EPS | MS |
| --- | --- | --- | --- | --- | --- |
| *Abies alba* | 1958-2018 | 4.73 (2.16) | 0.239 | 0.787 | 0.280 |
| *Abies grandis* | 1940-2017 | 4.36 (2.47) | 0.242 | 0.821 | 0.275 |
| *Abies veitchii* | 1979-2018 | 3.00 (1.72) | 0.405 | 0.881 | 0.390 |
| *Chamaecyparis lawsoniana* | 1911-2017 | 3.21 (1.58) | 0.336 | 0.807 | 0.360 |
| *Cryptomeria japonica* | 1969-2018 | 3.20 (1.82) | 0.443 | 0.901 | 0.406 |
| *Larix kaempferi* | 1945-2018 | 2.93 (1.48) | 0.607 | 0.953 | 0.450 |
| *Picea abies* | 1969-2018 | 3.99 (1.58) | 0.426 | 0.907 | 0.277 |
| *Pinus armandii* | 1981-2018 | 2.97 (1.30) | 0.322 | 0.874 | 0.255 |
| *Pinus nigra* | 1945-2018 | 2.44 (1.18) | 0.302 | 0.867 | 0.264 |
| *Picea omorika* | 1953-2018 | 2.75 (1.28) | 0.316 | 0.813 | 0.301 |
| *Picea orientalis* | 1944 -2017 | 3.03 (1.58) | 0.46 | 0.915 | 0.302 |
| *Picea sitchensis* | 1972 -2018 | 4.65 (1.65) | 0.279 | 0.848 | 0.222 |
| *Pseudotsuga menziesii* | 1916-2017 | 3.68 (1.69) | 0.402 | 0.867 | 0.311 |
| *Taxus baccata* | 1957-2018 | 2.15 (0.85) | 0.48 | 0.900 | 0.284 |
| *Taxus cuspidate* | 1974 2018 | 1.62 (0.71) | 0.341 | 0.834 | 0.252 |
| *Thuja plicata* | 1942-2017 | 4.02 (236) | 0.329 | 0.861 | 0.414 |
| *Tsuga canadensis* | 1972-2018 | 3.39 (1.59) | 0.315 | 0.751 | 0.199 |
| *Tsuga diversifolia* | 1972-2018 | 2.70 (1.24) | 0.435 | 0.884 | 0.278 |
| *Tsuga heterophylla* | 1971-2017 | 4.87 (2.20) | 0.282 | 0.850 | 0.284 |

Rbar indicates interseries correlation, EPS indicates expressed population signals, MS indicates sensitivity.

**Table S2** Species-specific *R^2^* was calculated from the square of correlations between the predicted tree ring index (based on the multiple regression in Table 2) and the observed tree ring index. *R^2^ind* indicates species-specific *R^2^* based on the individual level and *R^2^sp* indicates species-specific *R^2^* based on mean chronology (i.e., species) level.

| Species | *R^2^ind* | *R^2^sp* |
| --- | --- | --- |
| *Abies alba* | 0.044 | 0.121 |
| *Abies grandis* | 0.067 | 0.185 |
| *Abies veitchii* | 0.010 | 0.019 |
| *Chamaecyparis lawsoniana* | 0.080 | 0.173 |
| *Cryptomeria japonica* | 0.196 | 0.368 |
| *Larix kaempferi* | 0.226 | 0.304 |
| *Pinus armandii* | 0.054 | 0.089 |
| *Pinus nigra* | 0.141 | 0.280 |
| *Picea abies* | 0.199 | 0.360 |
| *Picea omorika* | 0.195 | 0.460 |
| *Picea orientalis* | 0.172 | 0.268 |
| *Picea sitchensis* | 0.197 | 0.372 |
| *Pseudotsuga menziesii* | 0.134 | 0.262 |
| *Taxus baccata* | 0.277 | 0.524 |
| *Taxus cuspidate* | 0.112 | 0.197 |
| *Thuja plicata* | 0.022 | 0.050 |
| *Tsuga canadensis* | 0.087 | 0.185 |
| *Tsuga diversifolia* | 0.107 | 0.207 |
| *Tsuga heterophylla* | 0.167 | 0.336 |
